# Gas Chromatography–Mass Spectrometry Metabolite Analysis Combined with Transcriptomics Reveals Genes Involved in Wax Biosynthesis in *Allium fistulosum* L.

**DOI:** 10.3390/ijms25116106

**Published:** 2024-06-01

**Authors:** Jiayi Xing, Huanhuan Xu, Mingzhao Zhu, Yuchen Zhang, Mifeng Bai, Xuyang Zhou, Huiying Liu, Yongqin Wang

**Affiliations:** 1 Beijing Vegetable Research Center, Beijing Academy of Agricultural and Forestry Sciences (BAAFS), Beijing 100097, China; jiayix2021@163.com (J.X.); 2022204057@stu.njau.edu.cn (H.X.); zhumingzhao@nercv.org (M.Z.); 15076753571@163.com (Y.Z.); baimifeng@163.com (M.B.); zhouxuyang@163.com (X.Z.); 2 Key Laboratory of Biology and Genetics Improvement of Horticultural Crops (North China), Ministry of Agriculture, Beijing 100097, China; 3 Beijing Key Laboratory of Vegetable Germplasms Improvement, Beijing 100097, China; 4 Key Laboratory of Special Fruits and Vegetables Cultivation Physiology and Germplasm Resources Utilization of Xinjiang Production and Construction Crops, Department of Horticulture, Agricultural College, Shihezi University, Shihezi 832003, China; 5 State Key Laboratory of Crop Genetics and Germplasm Enhancement and Utilization, College of Horticulture, Nanjing Agricultural University, Nanjing 210095, China

**Keywords:** *Allium fistulosum* L., gas chromatography–mass spectrometry, transcriptomics, wax biosynthesis, mutant

## Abstract

Cuticular waxes are essential for protecting plants from various environmental stresses. *Allium fistulosum* serves as an excellent model for investigating the regulatory mechanisms underlying cuticular wax synthesis with notable epidermal wax characteristics. A combination of gas chromatography–mass spectrometry (GC–MS) metabolite analysis and transcriptomics was used to investigate variations in metabolites and gene expression patterns between the wild type (WT) and glossy mutant type (*gl2*) of *A. fistulosum*. The WT surface had a large number of acicular and lamellar waxy crystals, whereas the leaf surface of *gl2* was essentially devoid of waxy crystals. And the results revealed a significant decrease in the content of 16-hentriacontanone, the principal component of cuticular wax, in the *gl2* mutant. Transcriptomic analysis revealed 3084 differentially expressed genes (DEGs) between WT and *gl2*. Moreover, we identified 12 genes related to fatty acid or wax synthesis. Among these, 10 DEGs were associated with positive regulation of wax synthesis, whereas 2 genes exhibited negative regulatory functions. Furthermore, two of these genes were identified as key regulators through weighted gene co-expression network analysis. Notably, the promoter region of *AfisC5G01838* (*AfCER1-LIKE1*) exhibited a 258-bp insertion upstream of the coding region in *gl2* and decreased the transcription of the *AfCER1-LIKE1* gene. This study provided insights into the molecular mechanisms governing cuticular wax synthesis in *A. fistulosum*, laying the foundation for future breeding strategies.

## 1. Introduction

The outer layer of the plant’s above-ground parts is covered by a hydrophobic cuticle, serving as a protective barrier against diverse external environmental threats [1,2,3]. This cuticle primarily consists of cuticular waxes and cutin [4,5]. The composition and quantity of waxes are crucial factors determining a plant’s ability to withstand various biotic and abiotic challenges [6]. Cuticular wax components encompass very-long-chain fatty acids (VLCFAs) and their derivatives [7], which amass to create various structures such as smooth, tubular, needle-like, and other crystal structures on the plant surface [8]. The wax formation involves two primary processes. First, the fatty acid synthase complex synthesizes C16 or C18 long-chain fatty acids de novo in the plastid. These C16 or C18 fatty acids are then elongated into C20–C34 VLCFA in the endoplasmic reticulum (ER). The resulting VLCFA can be converted into various compounds, such as aldehydes, alkanes, secondary alcohols, and ketones, through the alkane formation pathway, or primary alcohols and esters through the alcohol formation pathway [6]. The regulation of epidermal wax formation involves several genes. For instance, the content of the C29 wax component obtained from the *BrKCS6* mutant lines induced by EMS was significantly reduced [9]. Mutations in *BoCER2* resulted in a decrease in the wax content of cabbage below C28 [10]. The occurrence of smooth fruit mutants in blueberries can be attributed to a decrease in triterpenoid proportion during fruit development [11]. Mutant cabbage significantly decreased alkane content, whereas the WT demonstrated higher aldehyde content [12].

Different plant species and varieties exhibit variations in the composition and quantity of epidermal waxes, leading to differences in plant’s adaptation to distinct environments [13]. In *Agave*, for instance, alkanes are the main waxy components, with their content varying among different ploidy levels [14]. A significant reduction was observed in the content of 16-hydroxyhexadecanoic acid in the barley mutant *Cer-GN1* [15]. However, the *Allium cepa* mutant exhibited considerably reduced 16-hentriacontanone content, resulting in the absence of needle-like waxy crystals [16]. A wheat mutant was characterized by a reduced β-diketone content [17], and the lack of *BoCER2* in Chinese cabbage led to a sudden decrease in VLCFA content (C > 28) [18]. Creating novel mutants of *Dianthus spiculifolius* provided deeper insights into the function of cuticular waxes in drought resistance, with plants possessing increased cuticular wax displaying enhanced resistance compared with wild type (WT) [19]. The overexpression of the genes related to wax synthesis, such as *SlCER1-1* (tomato) [20], *PtoMYB142* (poplar) [9], and *TaCER1-6A* (wheat) [21], has been shown to enhance plant drought tolerance and total wax content. In addition, the cuticular wax layer plays a crucial role in plant insect resistance [22]. The overexpression of *CER3*, a cuticular wax synthesis gene from *Arabidopsis*, in cotton has been found to increase plant resistance to cotton whitefly [23]. Maize glossy mutants (*gl6*) exhibited reduced epicuticular wax content and plant drought tolerance, and increased cuticle permeability compared with the WT [24].

*Allium fistulosum* is a biennial herb of the *Allium* genus that is a major vegetable export of China [25]. Known for its antiseptic, anticancer, and antioxidant properties, *A. fistulosum* has garnered considerable acceptance among individuals seeking medicinal benefits [26,27]. Despite extensive research on the wax content and composition in related *Allium* species such as leeks [28,29], onions [16,30], and Welsh onions [31,32], the mechanisms underlying wax synthesis in *A. fistulosum* remain poorly understood. The present study aimed to identify a new wax deficiency mutant, followed by a systematic study of the key genes regulating cuticular wax structure, wax composition, and wax synthesis in the WT and mutant (*gl2*). This investigation provided valuable insights into understanding the essential genes involved in the cuticular wax biosynthesis pathway and metabolic processes in *A. fistulosum*.

## 2. Results

### 2.1. Phenotypic Observations, Wax Content Determination, and Wax Microstructure Observations of WT and gl2

**Figure 1:**
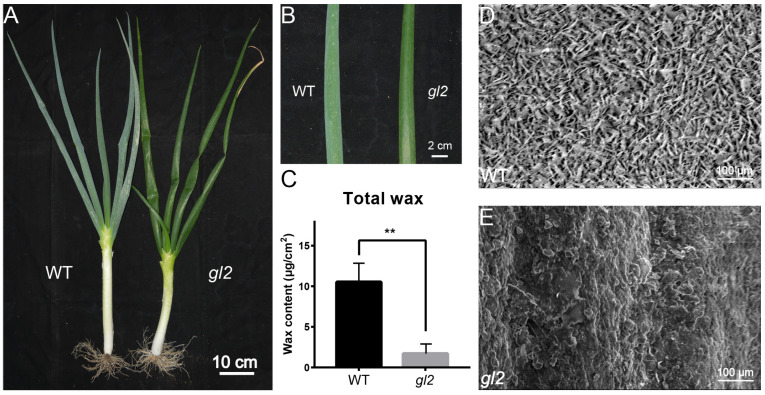
Phenotypic observations, wax content determination, and scanning electron microscope observations of WT and *gl2*. (**A**) Comparison of WT (left) and *gl2* (right) phenotypes. (**B**) Comparison of localized leaf phenotypes between WT (left) and *gl2* (right). (**C**) Total wax loading (μg/cm^2^) on the leaves of WT and *gl2*. Scanning electron microscopic observation of WT (**D**) and *gl2* (**E**) of *A. fistulosum* (magnification, 2000×). ** indicate the significance levels at *p* < 0.01.

Overall, no notable distinctions were observed in plant height, leaf spread, and leaf shape between the WT and *gl2* (Figure 1). The *gl2* plants were identified by their smooth, bright green leaves, whereas the WT plants had a white frost wax layer on the leaf surface. The analysis of wax content revealed that the total wax content of WT (10.52 ± 2.32 μg/cm^2^) was significantly higher than that of *gl2* (1.704 ± 1.19 μg/cm^2^).

Scanning electron microscopy (SEM) observations showed waxy crystal deposition on the leaf surfaces of both WT and *gl2* plants (Figure 1D,E). Specifically, the WT phenotype exhibited abundant waxy crystals with intricate acicular and flaky structures, whereas the leaf surface of *gl2* was largely devoid of waxy crystals.

### 2.2. Differences in Wax Composition between WT and gl2

**Figure 2:**
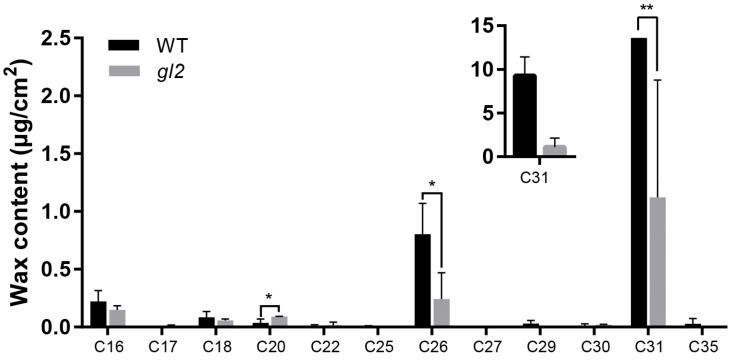
Wax composition of the leaf surface of WT and *gl2*. * and ** indicate the significance levels at *p* < 0.05 and *p* < 0.01, respectively.

To investigate differences in cuticular wax compounds between the WT and *gl2*, GC–MS was performed to determine the chemical composition of cuticular waxes (Figure 2). The results revealed that the wax compositions of both WT and *gl2* ranged from C16 to C35, with significant differences observed in certain wax compositions of *gl2* in terms of C20, C26, and C31 compared with WT. WT displayed a higher content of total wax components than gl2. Specifically, 16-hentriacontanone was identified as the main component of cuticular waxes in WT, which was significantly reduced in *gl2* (Table S2, Figure S1).

### 2.3. Analysis of Transcriptome Sequencing Results

RNA-seq analysis was conducted on three independent replicates of each sample (WT and *gl2*), acquiring 65.66 Gb of clean data using the *A. fistulosum* genome as a reference. The data from each sample ranged from 9.97 to 12.32 Gb, with a Q30 percentage of 94.43–95.33% and an average GC content of 43.69%, indicating suitability for downstream analysis (Table S3). The clean data were then compared and annotated, revealing 82,885 genes, including 4201 novel genes (Table S4).

### 2.4. Analysis of Differentially Expressed Genes

Numerous differentially expressed genes (DEGs) were identified across various groups, using the criteria of fold change (FC) ≥ 2 and false discovery rate (FDR) < 0.05 for DEG selection. A comparative analysis between WT and *gl2* revealed that 1441 genes were upregulated whereas 1643 genes were downregulated (Figure S2).

### 2.5. GO and KEGG Pathway Analyses

**Figure 3:**
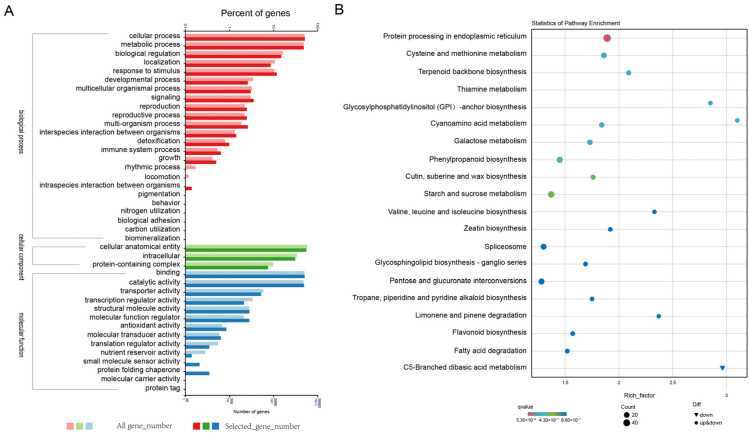
Analysis of DEGs of WT and *gl2*. (**A**) GO classification of the genes recognized within the biological process, molecular function, and cellular component categories. (**B**) Top 20 KEGG enrichment pathways of the DEGs.

GO annotation was conducted to explore the functions of the 3084 DEGs. The results revealed that 1326 DEGs (43%) were categorized under “cellular component,” 1632 DEGs (52.92%) under “molecular function,” and 2201 DEGs (56.14%) under “biological process” (Figure 3 and Table S5).

KEGG enrichment analysis was conducted to gain deeper insights into the DEGs associated with cuticular wax formation in *A. fistulosum* (Figure 3 and Table S6). The findings indicated significant enrichment of DEGs in pathways such as protein processing in the ER (ko04141), cysteine and methionine metabolism (ko00270), terpenoid backbone biosynthesis (ko00900), thiamine metabolism (ko00730), galactose metabolism (ko00052), phenylpropanoid biosynthesis (ko00940), cutin, suberine, and wax biosynthesis (ko00073), as well as fatty acid degradation (ko00071).

### 2.6. Genes Related to Fatty Acid and Wax Synthesis Pathways

**Figure 4:**
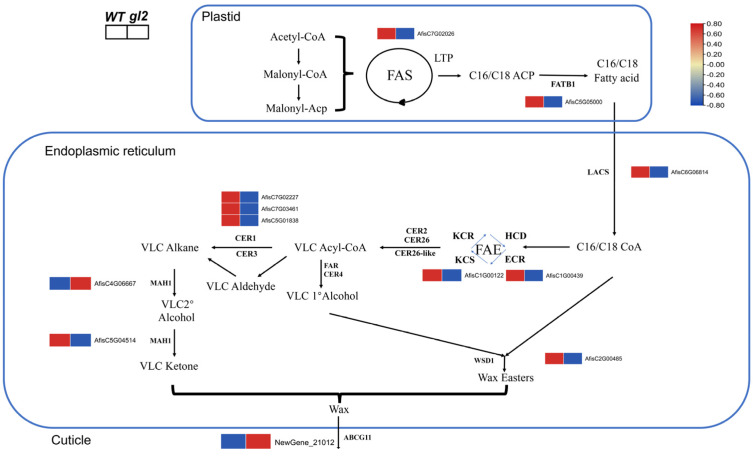
Metabolic pathways and clustering heat map of DEGs related to fatty acid and wax synthesis process. The color scale ranges from blue (low) to red (high), indicating the log_2_(FPKM + 1) values measured in WT (left) and *gl2* (right).

Twelve genes from DEGs, related to wax or fatty acid synthesis, were found to delineate the differences in the wax synthesis pathway between WT and *gl2* (Figure 4). Further analysis revealed that one DEG (*AfisC6G06814*; *LACS* homologue) participated in C16/C18 coenzyme A biosynthesis, two DEGs [*AfisC1G00122* and *AfisC1G00439*, members of fatty acid elongase (FAE) complex] were implicated in the biosynthesis of VLCFA, and three DEGs (*AfisC7G02227*, *AfisC7G03461*, and *AfisC5G01838*, homologues of *CER1-LIKE*) were involved in the biosynthesis of long-chain alkanes. Additionally, one DEG (*AfisC2G00485*; homologue of *WSD1*) was involved in the synthesis of wax esters, two DEGs [*AfisC4G06667* and *AfisC5G04514*; midchain alkane hydroxylase (*MAH*) homologues] were associated with the biosynthesis of long-chain secondary alcohols and ketones, and one DEG (*NewGene_21012*; *ABCG11* homologue) played a role in transporting the synthesized waxes to the epidermis.

### 2.7. Quantitative Reverse Transcription PCR Validated the Expression Pattern of DEGs in the Wax Biosynthetic Pathway

**Figure 5:**
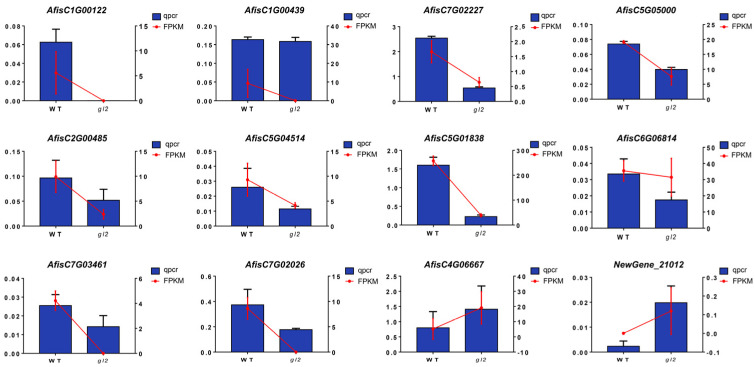
Validation of 12 DEGs in the leaves of *A. fistulosum* using qRT-PCR following detection by RNA-seq.

Twelve genes were selected for validation to confirm the credibility of DEGs identified in the transcriptome data. The expression of DEGs was verified in this pathway using qRT-PCR. The qRT-PCR results were consistent with the RNA-seq findings, showing a reasonable concurrence between the two techniques. Specifically, 10 DEGs involved in wax or fatty acid synthesis (*AfisC1G00122*, *AfisC1G00439*, *AfisC7G02227*, *AfisC5G05000*, *AfisC2G00485*, *AfisC5G04514*, *AfisC5G01838*, *AfisC6G06814*, *AfisC7G03461*, and *AfisC7G02026*) were found to be downregulated in *gl2*, whereas 2 DEGs associated with wax or fatty acid synthesis (*NewGene_21012* and *AfisC4G06667*) were upregulated in *gl2* (Figure 5).

### 2.8. Prediction of Transcription Factors

Transcription factors (TFs) are crucial regulators of signal transduction pathways in vivo. In this study, 2306 genes annotated as TFs were categorized into 67 families (Table S7). The analysis of the top 20 predicted TFs revealed that bHLH and MYB were the two most abundant TF families (Figure S3). The predicted TFs were screened for a fold change >1 and observed differences in Fragments Per Kilobase Million (FPKM) values, mainly for MYB, B3, bZIP, and WRKY (Table S8). Heatmaps were generated to illustrate the differences in the expression of TFs between WT and *gl2* (Figure S4).

### 2.9. WGCNA Analysis

**Figure 6:**
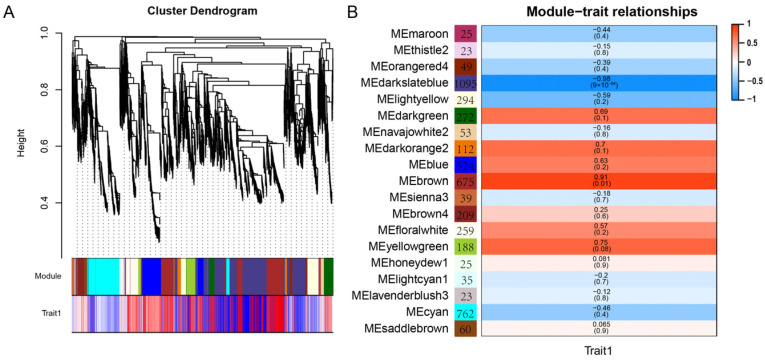
WGCNA analysis based on the transcriptional data of WT and *gl2*. (**A**) Clustering dendrogram illustrating co-expression modules identified by WGCNA. This dendrogram comprises 19 modules, distinguished by various colors. (**B**) Associations between modules and characteristics are depicted. Rows and columns correspond to modules and traits, respectively, with numbers in the module boxes representing gene counts. The colors of the cells where rows and columns intersect indicate the correlation coefficients between modules and traits. The color scale on the right side denotes the correlation coefficient, where red indicates a positive correlation and blue indicates a negative correlation. “Trait1” denotes the wax characteristic.

WGCNA was conducted for *A. fistulosum* genes to further explore genes potentially associated with wax synthesis, transportation, and regulation (Figure 6). The results revealed that WGCNA categorized genes into 19 modules, with sizes ranging from 23 (“MEthistle2” and “MElavenderblush”) to 1095 (“MEdarkslateblue”). A significant positive correlation was observed between the “brown” module and wax phenotype (gene significance 0.91, *p* < 0.05). Importantly, the key DEGs *AfisC7G02227* and *AfisC5G01838* (Figure 5) identified earlier were also found in “Brown” module, suggesting that these genes might play a key role in regulating wax synthesis.

### 2.10. Transcriptional Level of AfCER1-LIKE Decreased by Promoter Region Mutation

FPKM values, qPCR analysis of DEGs, and WGCNA analysis indicated that *AfisC5G01838* played a crucial role in wax synthesis. Evolutionary tree analysis showed that AfisC5G01838 and AfisC7G02227 were homologous to CER1 genes from other species, named AfCER1-LIKE1 and AfCER1-LIKE2, respectively (Figure S5).

**Figure 7:**
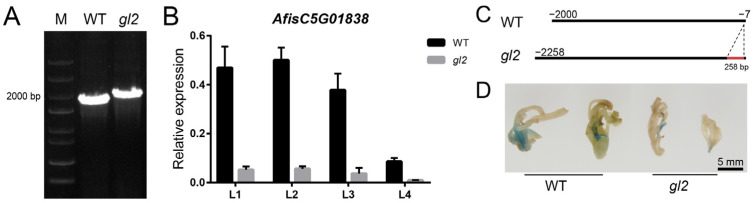
Variation of the promoter region of *AfCER1-LIKE1* and changes in expression in different leaves. (**A**) Agarose gel electrophoresis image of *AfCER1-LIKE1* in WT and *gl2* promoter sequences. M: Trans5K DNA Marker. (**B**) *AfCER1-LIKE1* expression in different leaves of WT and *gl2*. (L1: the first leaf, L2: the second leaf, L3: the third leaf, L4: the fourth leaf). (**C**) Schematic representation of *AfCER1-LIKE1* promoter sequences in WT and *gl2*; red line indicates the region added in *gl2*. (**D**) GUS staining of positive plants of tobacco transformed with promoter sequences of WT and *gl2* of *AfCER1-LIKE1*.

The coding region and promoter sequences of *AfCER1-LIKE1* were cloned to further investigate its function. The results revealed that *AfCER1-LIKE1* had the same coding region sequence in both WT and *gl2*, but a fragment of 258 bp insertion was observed 5′ upstream of the *gl2* coding region compared with that in WT (Figure 7 and Figure S6). GUS staining of transgenic tobacco plants showed that the promoter sequence activity of WT was higher than that of *gl2*. Meanwhile, the expression of *AfCER1-LIKE1* gene was significantly higher in WT than in *gl2*. *AfCER1-LIKE1* expression was highest in L1 and L2 and tended to decrease with increasing leaf position.

## 3. Discussion

The cuticle is attached to the surface of plants, enabling them to endure various environmental stresses [5]. A notable correlation was observed between cuticular waxes and cutins, with some genes sharing overlapping or similar functions [33]. The identification of waxy mutants has contributed to understanding the precise molecular mechanisms underlying epicuticular wax formation, offering valuable insights for agricultural breeding. Although several studies have focused on wax-deficient mutants in crops such as barley [15,34], wheat [35], maize [36], and rice [37]. Studies on this topic in *A. fistulosum* are limited. Previous studies by the present group have successfully identified BianGan Welsh onion varieties with low-temperature dormancy and examined the identification of wax-related genes as well as the physiological characteristics of the WT BianGan (BG) and the bright green BianGan Welsh onion (GLBG) [32]. In this study, a new wax-deficient mutant of *A. fistulosum* was identified and the DEGs associated with wax synthesis and components were investigated using transcriptional analysis and GC–MS. These findings lay the foundation for elucidating the mechanisms of epicuticular wax formation and offer the potential for breeding bright and glossy green Welsh onion varieties, which may be more appealing to consumers.

### 3.1. Cuticular Wax Composition

The SEM analysis revealed distinct differences in the morphology and quantity of waxy crystals on the leaf surfaces of WT and the glossy mutant of *A. fistulosum*. In WT, waxy crystals were densely distributed across the surface, exhibiting two primary forms. Conversely, *gl2* lacked waxy crystals, which was in agreement with the findings shown for barley [38]. Furthermore, the GC–MS analysis indicated a notably higher content of 16-hentriacontanone in the WT compared with *gl2*, which was consistent with wax composition trends identified in *A. cepa* [16]. These results underscored the remarkable variations in wax composition and content among different crop species. Varieties with different wax compositions also showed inconsistent resistance to biotic stresses. We observed that WT thrips was severely affected, while *gl2* was less so (Figure S7). This may be due to the different attractiveness of thrips to different wax compositions. This is consistent with the results that onions with a glossy phenotype showed resistance to thrips [39], and that this could be used as a breeding material to improve biotic stress resistance in *A. fistulosum*.

### 3.2. Identification of Key Genes Related to Wax Synthesis

Cuticular wax biosynthesis begins with the biosynthesis of precursor fatty acids within the plastid, which are later transported to the ER for elongation into VLCFAs (>C20). The cuticular wax primarily consists of VLCFA and their derivatives, including alkanes, ketones, esters, aldehydes, and primary and secondary alcohols [4]. The process of wax synthesis is complex and involves the coordinated control of several genes [16]. Any deletion or mutation in the involved genes may alter their function, leading to variations in the cuticular wax (Figure 4). For example, the mutations in *LACS1* in *Arabidopsis* reduce the amount of wax content in all chemical classes on the stem and leaves [40]. The key components of the FAE complex, such as 3-ketoacyl-CoA synthetases and enoyl-CoA reductase, catalyze the elongation of VLCFAs, influencing their chain length and enhancing drought stress tolerance in transgenic plants [41,42]. Genes such as *CER1* and *CER3* play pivotal roles in alkane synthesis in citrus fruit [43]. Furthermore, in sunflower, plant wax esters are crucial components of cuticular wax and their synthesis is catalyzed by diacylglycerol acyltransferase (*WSD*) [44]. *MAH* is implicated in wax biosynthesis. Some transcription factors such as *MdMYB106* and *MdDEWAX* affect the increase or decrease in wax content [45,46]. We analyzed the differentially expressed transcription factors to prepare for further screening of genes involved in wax synthesis (Figure S4).

Plants with mutant alleles with T-DNA insertions either lacked secondary alcohols and ketones (*mah1-1*) or exhibited reduced levels of secondary alcohols and ketones (*mah1-2* and *mah1-3*) in stem wax composition compared with the WT [47]. In the present study, 12 key DEGs were identified in the wax synthesis pathway of *A. fistulosum* that might play crucial roles in regulating wax or fatty acid synthesis. Among these, 10 upregulated DEGs positively regulated wax or fatty acid production, whereas 2 downregulated DEGs negatively regulated wax or fatty acid production. WGCNA analysis revealed that *AfCER1-LIKE1* and *AfCER1-LIKE2* (annotated as *CER1-LIKE* with different sequences in the coding region) (Figure S8) were significantly positively correlated with the wax phenotype. The CER1-LIKE evolutionary tree showed that AfCER1-LIKE1 and AfCER1-LIKE2 were homologous to other CER1-LIKE genes, indicating that AfCER1-LIKE1 and AfCER1-LIKE2 are CER1-LIKE genes, while AfCER1-LIKE1 is more closely related (Figure S5). *BdCER1-8* and *SlCER1-1* play a major role in the biosynthesis of VLC alkanes in leaves [20,48]. A base insertion in the *AfCER1-LIKE1* promoter sequence suggested that AfCER1-LIKE1 may be the main cause of the emergence of *gl2* mutants. GUS staining of transgenic tobacco plants showed that the promoter sequence activity of WT was higher than that of *gl2*, indicating that the 258 bp insertion did affect the expression of the AfCER1-LIKE1 gene. However, further investigation is needed to elucidate its specific role in the wax synthesis pathway (Figure 7 and Figure S6). The AfCER1-LIKE1and AfCER1-LIKE2 genes are redundant. In contrast, no differences were observed in the sequences of the AfCER1-LIKE2 coding and promoter regions between the WT and *gl2* varieties. The downregulation of *AfisC5G04514* might be associated with a substantial reduction in the composition of 16-hentriacontanone in *gl2*. Moreover, the inhibition of ketone synthesis likely led to substrate accumulation and subsequent upregulation of *AfisC4G06667* gene expression. However, further studies are needed to determine the exact functions of these genes within the pathway. *ABCG11* plays a crucial role in the normal formation of the stratum corneum and serves as a vital element in the keratin lipid export process. The knockout of *abcg11* led to a notable decrease in the alkane content in *Arabidopsis* [49,50]. ABCG11 could transport cutin monomers and wax components [51]. The upregulation of *AfCER1-LIKE1* and *AfCER1-LIKE2* may contribute to the cuticular wax synthesis, although further elucidation of their specific functions is required. The upregulation of *Newgene_21012* (annotated as *ABCG11*) in *gl2* may be attributed to the transport of cutin monomers to the epidermis. In this study, the heatmap of the wax metabolic pathway indicated that the expression levels of relevant genes involved in wax synthesis were higher in WT compared with *gl2* (Figure 4 and Figure 5). This suggested that 10 genes with reduced expression in *gl2* might play a positive regulatory role in wax synthesis. In contrast, two genes, *NewGene_21012* and *AfisC4G06667*, with increased expression in *gl2*, might have a negative impact on wax synthesis regulation.

## 4. Materials and Methods

### 4.1. Plant Materials

The mutants of *A. fistulosum* with less waxed leaf were isolated from CSC varieties and named *gl2*. The plant was cultivated at the Yanqing Experimental Base of the Vegetable Research Center, Beijing Academy of Agriculture and Forestry Sciences, Beijing, China. This experiment used *A. fistulosum* plants grown for about 6 months as material. For simplicity, the WT and mutant of *A. fistulosum* are referred to as WT and *gl2*, respectively, in the following text. And the materials were provided by Yongqin Wang.

### 4.2. Scanning Electron Microscopy

Fresh leaves of *A. fistulosum* were collected and washed with distilled water to eliminate any surface debris. The leaves were sliced into 5 × 5 mm^2^ pieces and dried in an oven. Then, the specimens were mounted on the sample platform using a conductive adhesive and coated with a layer of gold. The leaf wax was examined using a Hitachi S-3400N scanning electron microscope (Hitachi, Tokyo, Japan).

### 4.3. Extraction of Leaf Cuticle Wax and Determination of Wax Content

The total wax was extracted from the third leaf outward from the growth point of *A. fistulosum*. The middle portion of each undamaged whole leaf was washed and blown dry using distilled water and then immersed in chloroform for 15 s and removed. The leaf area was calculated using Image J (v. 1.54i). Subsequently, 25 µL (1 µg/µL) of *n*-tetracosane (C_24_) was added as an internal standard reference. The chloroform was evaporated using a nitrogen stream, following which 100 μL of N,O-*bis* (trimethylsilicyl) fluoroacetamide and 100 μL of pyridine were added for derivatization in an incubator at 70 °C for 60 min. After nitrogen drying, 300 μL of *n*-hexane was added to dissolve the sample, which was then filtered. The wax composition was analyzed using an Agilent 7890B-5977A gas chromatography–mass spectrometry (GC–MS) instrument (Agilent technology, Santa Clara, CA, USA). The gas chromatographic conditions were an HP-5 (30 m × 0.25 mm × 0.25 μm) quartz capillary column with an inlet temperature of 250 °C and a constant flow rate of 1.1 mL/min; an ion source temperature of 230 °C, an Aux-2 temperature of 290 °C, a quadrupole temperature of 150 °C, and helium as the carrier gas, with a non-split injection and a sample volume of 1 μL. The GC–MS procedure involved an initial temperature of 80 °C for 2 min, followed by a temperature increase to 290 °C at a rate of 4 °C/min, and maintained for 20 min. The wax compositions were identified by analysis in the NIST14 database. All wax components were quantified by comparison with the peak area of the internal standard n-tetracosane. In contrast, the wax constituent amounts were expressed in the form of per unit of leaf area. Each material was replicated three times, with each replicate consisting of five leaves.

### 4.4. RNA Sequencing and Transcriptome Data Analysis

The leaves were promptly frozen in liquid nitrogen after collection and then stored at −80 °C in the refrigerator. Plant total RNA was extracted using an RNAprep Pure Plant Kit (Tiangen, Beijing, China). The raw data were processed, and readings containing adapters, ploy-N sequences, and low-quality sequences were discarded to obtain clean data. The raw sequence data generated were deposited in the Sequence Read Archive (SRA) database at National Center for Biotechnology Information (NCBI) under the Accession number PRJNA1065148. RNA-seq analysis was conducted by Biomarker Technologies (Beijing, China) using the Illumina NovaSeq6000 sequencing platform (San Diego, CA, USA). The expression levels were quantified using FPKM values. The WT and *gl2* were compared using WT as the baseline to assess gene up- and downregulation and use TBtools to plot a heat map [52]. And the color scale was determined using the log_2_(FPKM + 1) values of WT (left) and *gl2* (right) to standardize the data.

### 4.5. Expression Analysis of the Candidate Genes Using Real-Time Polymerase Chain Reaction

Gene expression was analyzed as follows: The cDNA was synthesized by HiScript III All-in-One RT SuperMix Perfect for quantitative polymerase chain reaction (qPCR) (Vazyme, Nanjing, China). Primers used in this study were provided in Table S1. The qPCR reaction was followed by the instrument of 2× SYBR qPCR Master Mix (Vazyme) on Roche lightcycler 480 (Roche, Basel, Switzerland). The relative expression of genes was calculated by the 2^−ΔΔCt^ method [53].

### 4.6. GUS Staining of Transgenic Tobacco

The *AfisC5G01838* promoter sequence fragments of WT and *gl2* were respectively attached to the pBI101 vector (XbaⅠ (Takara, Kyoto, Japan) single enzyme digestion). The correctly sequenced recombinant plasmid was transferred into the susceptible state of *Agrobacterium* GV3101 (Weidi biotechnology, Shanghai, China) by heat shock method, and then *Agrobacterium* identified by PCR as a positive clone was impregnated into tobacco leaves by leaf disk method. The differentiated transgenic positive plants were treated with GUS staining kit (Solarbio, Beijing, China) at 37 °C overnight, and then decolorized with 75% ethanol to observe the staining.

### 4.7. Statistical Analysis

Statistical analyses were conducted using GraphPad Prism 7 software following the method outlined by Mitteer et al. (2018) [54]. Data were statistically analyzed using one-way ANOVA, with *p* ≤ 0.05 indicating significant differences.

## 5. Conclusions

In this study, we preliminarily investigated the cause of the formation of less-wax mutant *gl2* of *A. fistulosum*. The wax compound analysis exhibited decreased in the content of 16-hentriacontanone in *gl2*. Transcriptome analysis showed that *AfCER1-LIKE1* and *AfCER1-LIKE2* may have a critical role in modulating wax synthesis. Furthermore, the promoter region of *AfCER1-LIKE1* exhibited a 258-bp insertion upstream of the coding region in *gl2* and decreased the transcription of the *AfCER1-LIKE1*. In conclusion, this study provided theoretical insights into the molecular mechanisms underlying *A. fistulosum* cuticular wax synthesis and contributed to agricultural breeding efforts.

## Data Availability

All data are included in the article. RNA-seq raw data are available at the NCBI database (https://www.ncbi.nlm.nih.gov/bioproject/?term=PRJNA1065148 (accessed on 16 January 2024).

## Supplementary Materials

The following supporting information can be downloaded at: https://www.mdpi.com/article/10.3390/ijms25116106/s1.
