# Supplementary material for: Gas Chromatography–Mass Spectrometry Metabolite Analysis Combined with Transcriptomics Reveals Genes Involved in Wax Biosynthesis in Allium fistulosum L
Source: Int J Mol Sci. 2024 Jun 1;25(11):6106. doi: 10.3390/ijms25116106 (PMC11173144; doi:10.3390/ijms25116106)
Supplement: Supplementary file 1 [file ijms-25-06106-s001.zip › Supplementary Figures.pdf]

Supplementary Figures

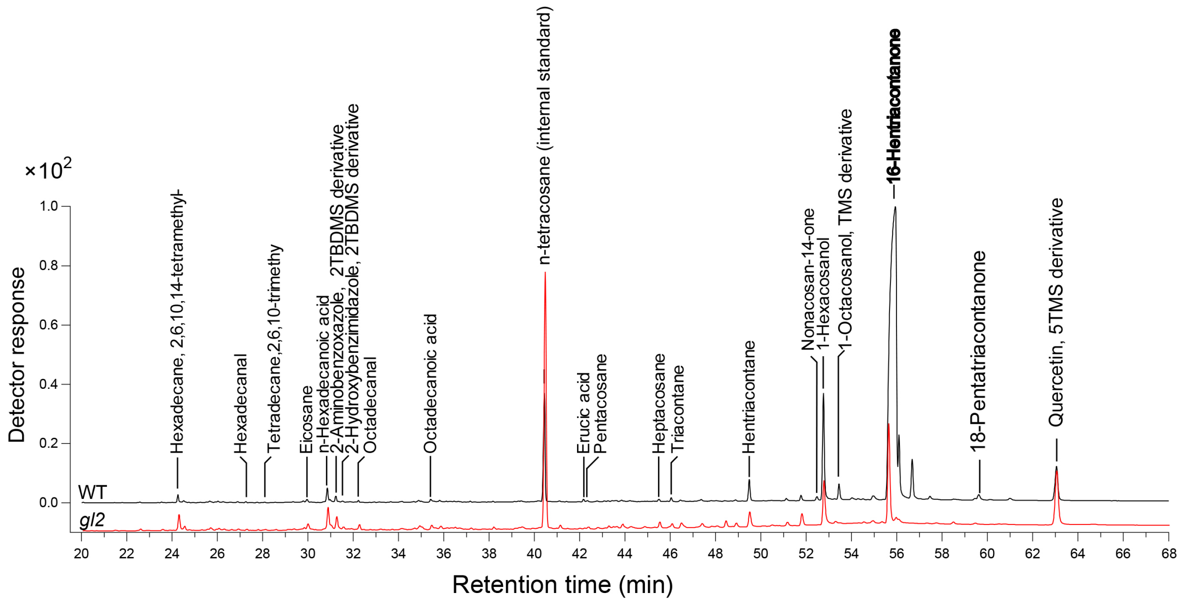

Figure S1. Total ion current chromatogram of the separation of components of epicuticular waxes of *Allium fistulosum* L. (WT: black; *gl2*: red.).

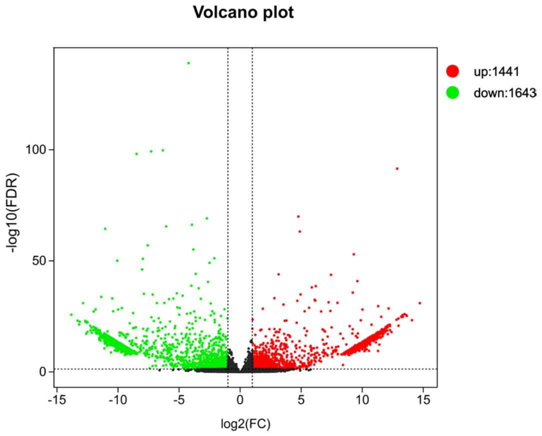

Figure S2. Volcano map of differentially expressed genes.

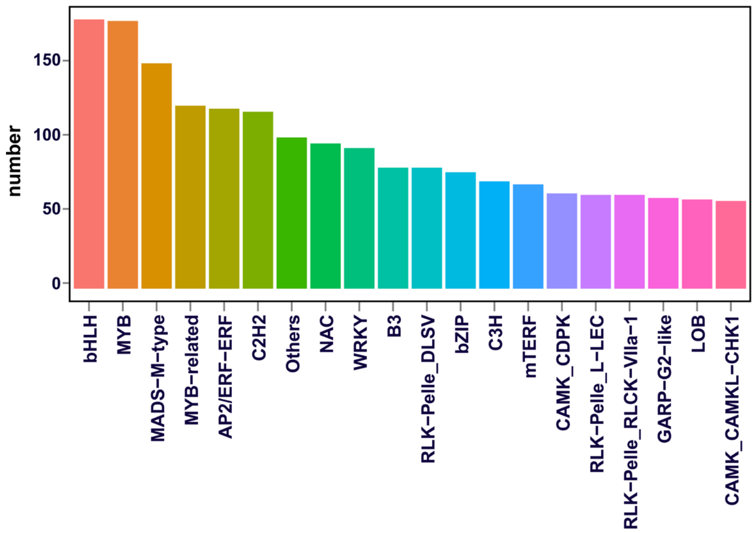

Figure S3. Prediction of transcription factors

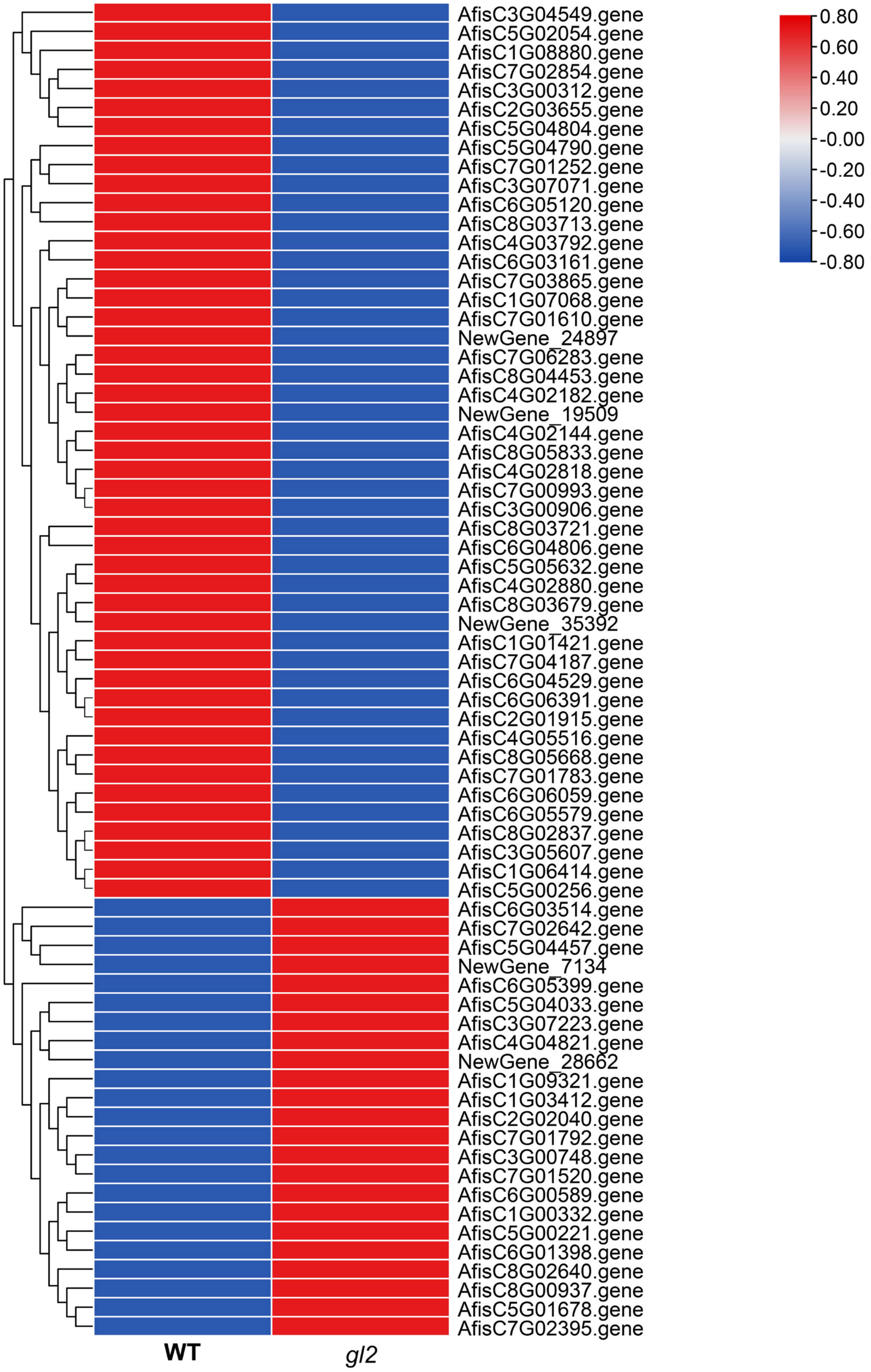

Figure S4. Transcription factor genes differentially expressed in WT and *gl2*. TBtools was used to produce heat maps. The color scale ranges from blue (low) to red (high), indicating the $\log_2$(FPKM + 1) values measured in WT and *gl2*.

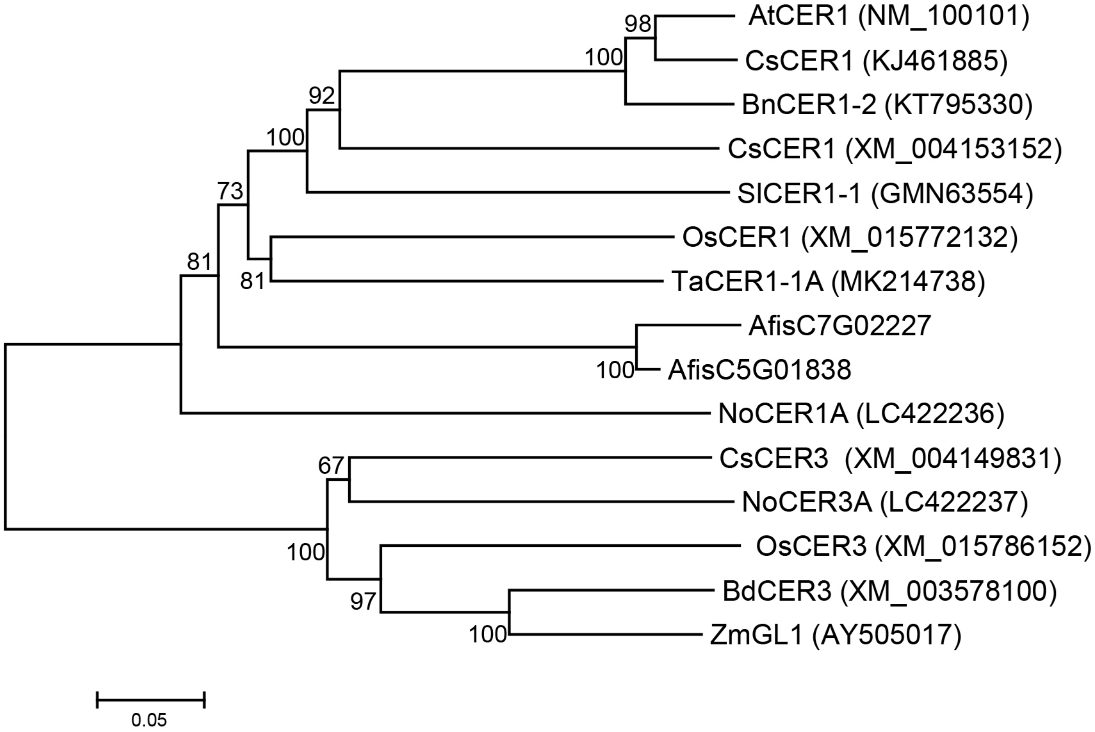

Figure S5. Evolutionary tree analysis of CER1-LIKE between *Allium fistulosum* (AfisC5G01838 and AfisC7G02227) and other species. At, *Arabidopsis thaliana*; Cs, *Camelina sativa*; Bn, *Brassica napus*; Sl, *Solanum lycopersicum*; Os, *Oryza sativa*; Ta, *Triticum aestivum*; No, *Nymphaea odorata*; Bd, *Brachypodium distachyon*; Zm, *Zea mays*. MEGA 6 was used to build the evolutionary tree.

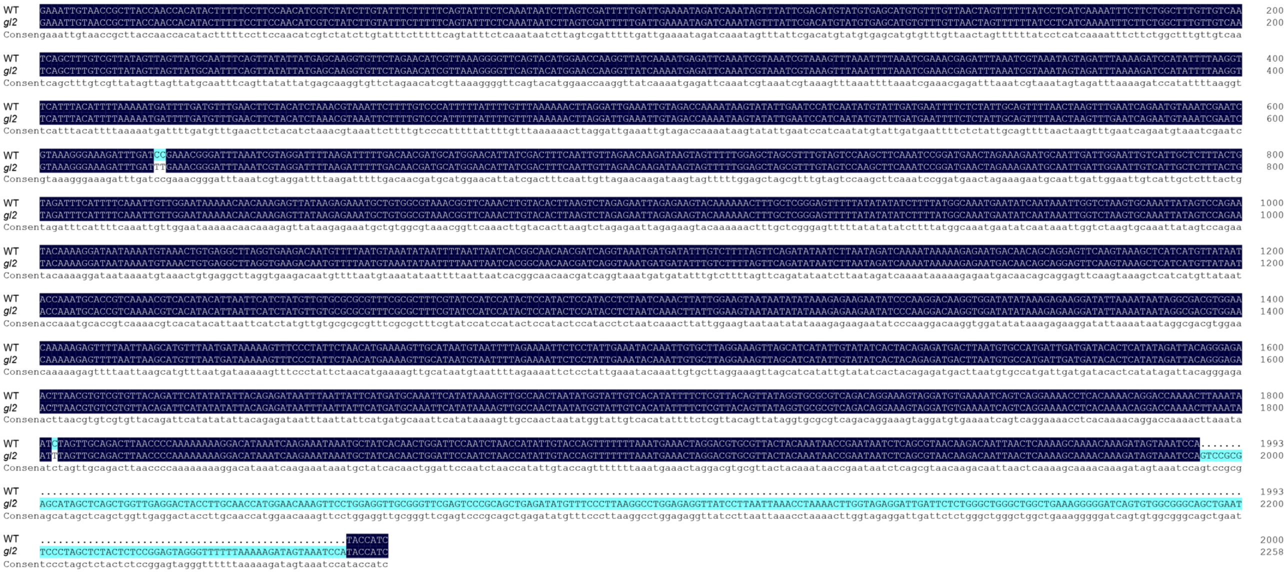

Figure S6. *AfisC5G01838* gene promoter sequence comparison of WT and *gl2*.

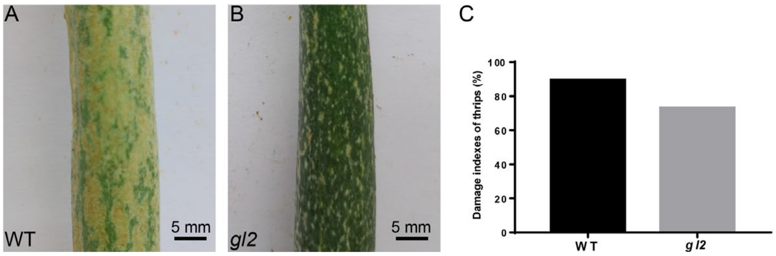

Figure S7. Picture of thrips biting on plants WT (A) and gl2 (B). C: Statistics on the thrip damage index.

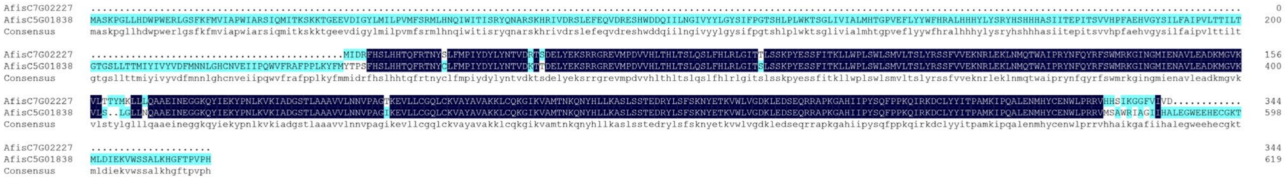

Figure S8. Sequence alignment of *AfisC5G01838* and *AfisC7G02227* amino acids.
